# TET2-interacting long noncoding RNA promotes active DNA demethylation of the MMP-9 promoter in diabetic wound healing

**DOI:** 10.1038/s41419-019-2047-6

**Published:** 2019-10-25

**Authors:** Liyan Zhou, Meng Ren, Tingting Zeng, Wei Wang, Xiaoyi Wang, Mengdie Hu, Shicheng Su, Kan Sun, Chuan Wang, Jing Liu, Chuan Yang, Li Yan

**Affiliations:** 1 Department of Endocrinology, Sun Yat-sen Memorial Hospital, Sun Yat-sen University, 107 Yanjiang West Road, Guangzhou, 510120 China; 2 Guangdong Provincial Key Laboratory of Malignant Tumor Epigenetics and Gene Regulation, Sun Yat-sen Memorial Hospital, Sun Yat-sen University, 107 Yanjiang West Road, Guangzhou, 510120 China; 3 Department of phase I Clinical Trial, Tianjin Medical University Cancer Institute and Hospital, Tianjin Medical University, Huanhu West Road, Tianjin, 300060 China; 4 Breast Tumor Center, Sun Yat-sen Memorial Hospital, Sun Yat-sen University, 107 Yanjiang West Road, Guangzhou, 510120 China

**Keywords:** DNA methylation, Long non-coding RNAs

## Abstract

Wound healing in diabetic skin is impaired by excessive activation of matrix metalloproteinase-9 (MMP-9). MMP-9 transcription is activated by Ten-eleven translocation 2 (TET2), a well-known DNA demethylation protein that induces MMP-9 promoter demethylation in diabetic skin tissues. However, how TET2 is targeted to specific loci in the MMP-9 promoter is unknown. Here, we identified a TET2-interacting long noncoding RNA (TETILA) that is upregulated in human diabetic skin tissues. TETILA regulates TET2 subcellular localization and enzymatic activity, indirectly activating MMP-9 promoter demethylation. TETILA also recruits thymine-DNA glycosylase (TDG), which simultaneously interacts with TET2, for base excision repair-mediated MMP-9 promoter demethylation. Together, our results suggest that the TETILA serves as a genomic homing signal for TET2-mediated demethylation specific loci in MMP-9 promoter, thereby disrupting the process of diabetic skin wound healing.

## Introduction

Diabetic foot ulcers (DFUs) are major complication of diabetes^1,2^. High blood sugar triggers prolonged chronic inflammation with concomitant elevated levels of matrix metalloproteinases (MMPs) in diabetic patients. MMPs are a family of a zinc-dependent endopeptidase family that degrades extracellular matrix (ECM) components involved in tissue remodeling^3^. The excess protease activity can lead to delay diabetic wound healing and result in limb amputation, especially matrix metalloproteinase-9 (MMP-9), which was present in more than 50% of the chronic wounds^4–6^. Many studies show that elevated MMP-9 expression contributes to delayed wound healing and high quantities of bacteria in the wound, while decreased MMP-9 expression promotes diabetic wound healing^7–9^.

Increasing studies have revealed that MMP-9 expression is critically mediated by epigenetic mechanisms, including histone modification, DNA methylation, and noncoding RNA^5,10^. Advanced glycation end-products (AGEs) are generated from non-enzymatic and irreversible reactions between reducing sugars and amino groups of proteins under hyperglycemic environment. It is an important approach to use AGEs to mimic diabetic conditions in vitro, especially the epigenetic mechanisms of diabetic complication^11,12^. Recently, we published that AGEs induce binding of the protein Ten-eleven translocation 2 (TET2) to the MMP-9 promoter and impair diabetic wound healing^13^. However, the molecular mechanisms underlying how TET2 is targeted to MMP-9 promoter-specific loci in diabetic skin cells remain unresolved.

Long noncoding RNA (lncRNA) is a large class of non-protein-coding transcript greater than 200 bases in length that is involved in numerous physiological and pathological processes^14^. Recent data suggest that lncRNA modulates cell signaling pathways via interactions with protein partners^15–18^. Specifically, lncRNA can act as modular scaffolds to regulate chromatin states and epigenetic inheritance^19,20^. For example, Ruscio et al. identified a functional lncRNA (*ecCEBPA*) that interacted with DNA methyltransferase 1 (DNMT1) and prevented gene locus methylation through chromatin level regulation^21^. However, whether demethylation enzymes like TET2 also interact with lncRNA to target specific promoters are unknown.

In this study, we identified a TET2-interacting lncRNA (TETILA) that activates MMP-9 transcription by inducing TET2 dependent DNA demethylation. We showed that TETILA recruits TET2 and thymine-DNA glycosylase (TDG) to form a demethylation complex at the MMP-9 promoter, ultimately increasing MMP-9 expression. Thus, this lncRNA serves as homing signal for gene-specific DNA demethylation in TET2-mediated epigenetic regulation and participates in delayed diabetic wound healing.

## Results

### Identification of a TET2-binding lncRNA

**Fig. 1:**
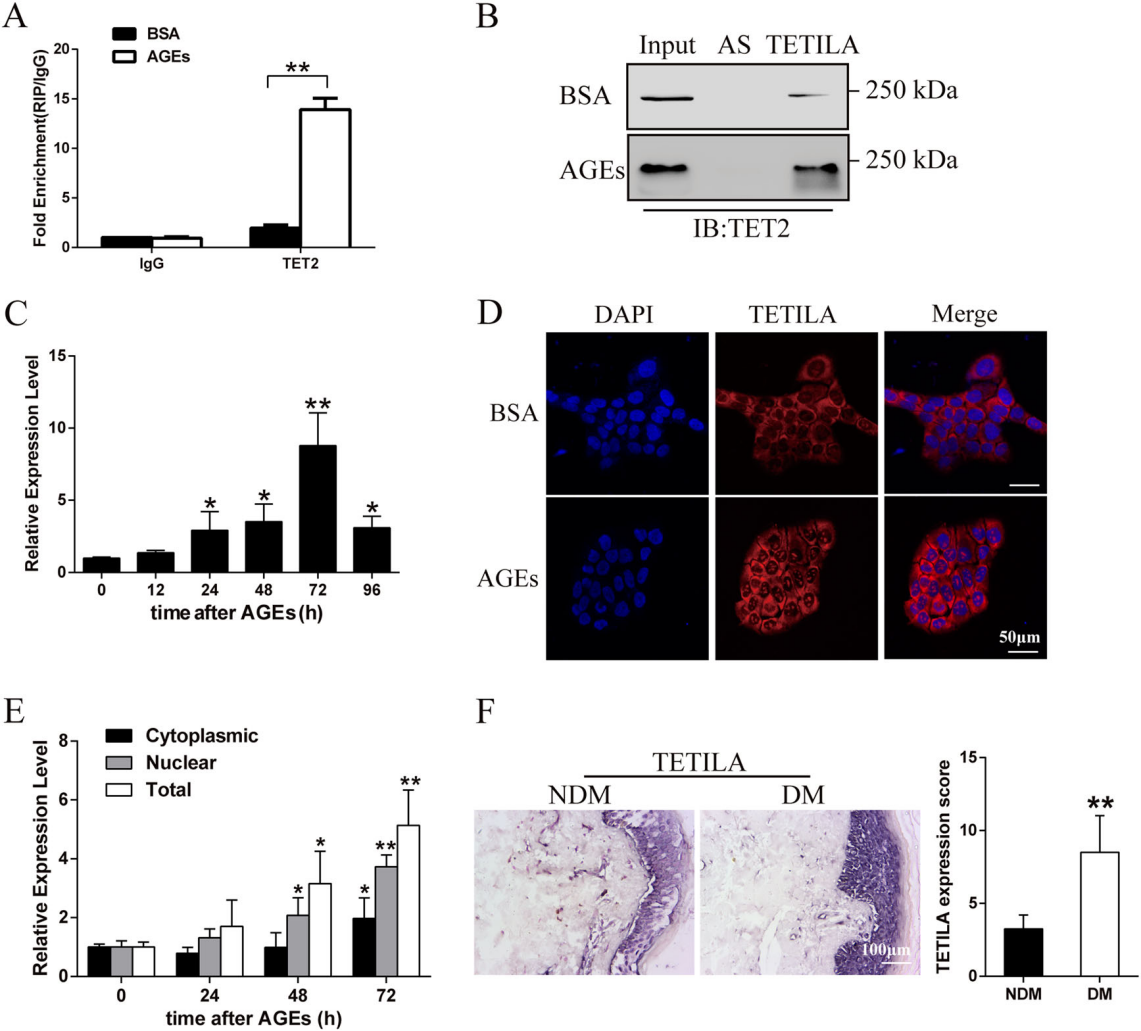
Characterization of TETILA expression. **a** Relative RIP assays using qPCR to detect binding between TETILA and TET2 in BSA- and AGEs-treated HaCaT cells (*P* = 0.000). **b** RNA pull-down showing the interaction between TETILA and TET2 in BSA- and AGEs-treated HaCaT cells. **c** TETILA expression kinetics in HaCaT cells following AGEs stimulation. **d** Confocal FISH images showing nuclear and cytoplasmic localization of TETILA in HaCaT cells. **e** TETILA expression measured by RT-qPCR in nuclear and cytoplasmic fractionations under BSA or AGEs treatment. **f** The expression of TETILA in foot skin from diabetic patients as measured by ISH (*P* = 0.008). The histograms indicate the quantitative analysis of TETILA expression in patients skin tissues. All PCR data were normalized to ACTB expression and represented as mean ± SD from three independent experiments. *P *<* 0.05, **P *<* 0.01 vs. the corresponding control group

We first analyzed different lncRNA expression patterns, which bound to TET2 protein between bovine serum albumin (BSA)- and AGEs-treated group in HaCaT cells (Fig. S1a). Using a 5-fold cut-off threshold and deleting lncRNAs with low raw intensities, we extracted 48 lncRNAs that were differentially expressed between the two treatment groups (Table S1). We found lncRNA-G072813 (named TET2- interacting lncRNA, TETILA) was the highest enriched in TET2-RNA precipitates (Fig. 1a). We further demonstrated a physical interaction between TETILA and TET2 using RNA pull-down assay (Fig. 1b). TETILA was predicated to be noncoding using open reading frames (ORF) and coding potential calculator software (Fig. S1b), and it was a 2564 nucleotide (nt) transcript in human chromosome 6 and contained three exons (Table S2 and Fig. S1c).

In HaCaT cells, TETILA was robustly upregulated beginning after AGEs stimulation (Fig. 1c). RNA-fluorescence in situ hybridization (RNA-FISH) and cellular fractionation assay revealed that this lncRNA was expressed in both the nucleus and cytoplasm of HaCaT cells and primary human keratinocytes (Fig. 1d, Fig. S1d and Fig. S3e-f). Interestingly, AGEs treatment significantly increased lncRNA nuclear and cytoplasm expression relative to zero time point (Fig. 1e). We found that TETILA expression levels were significantly higher in diabetic skin tissues relative to non-diabetic ones (Fig. 1f). We then observed TETILA expression in different human tissues using in situ hybridization (ISH) and found it was the highest in the skin relative to other tissues (Fig. S1e).

### TETILA enhances TET2 protein stability

**Fig. 2:**
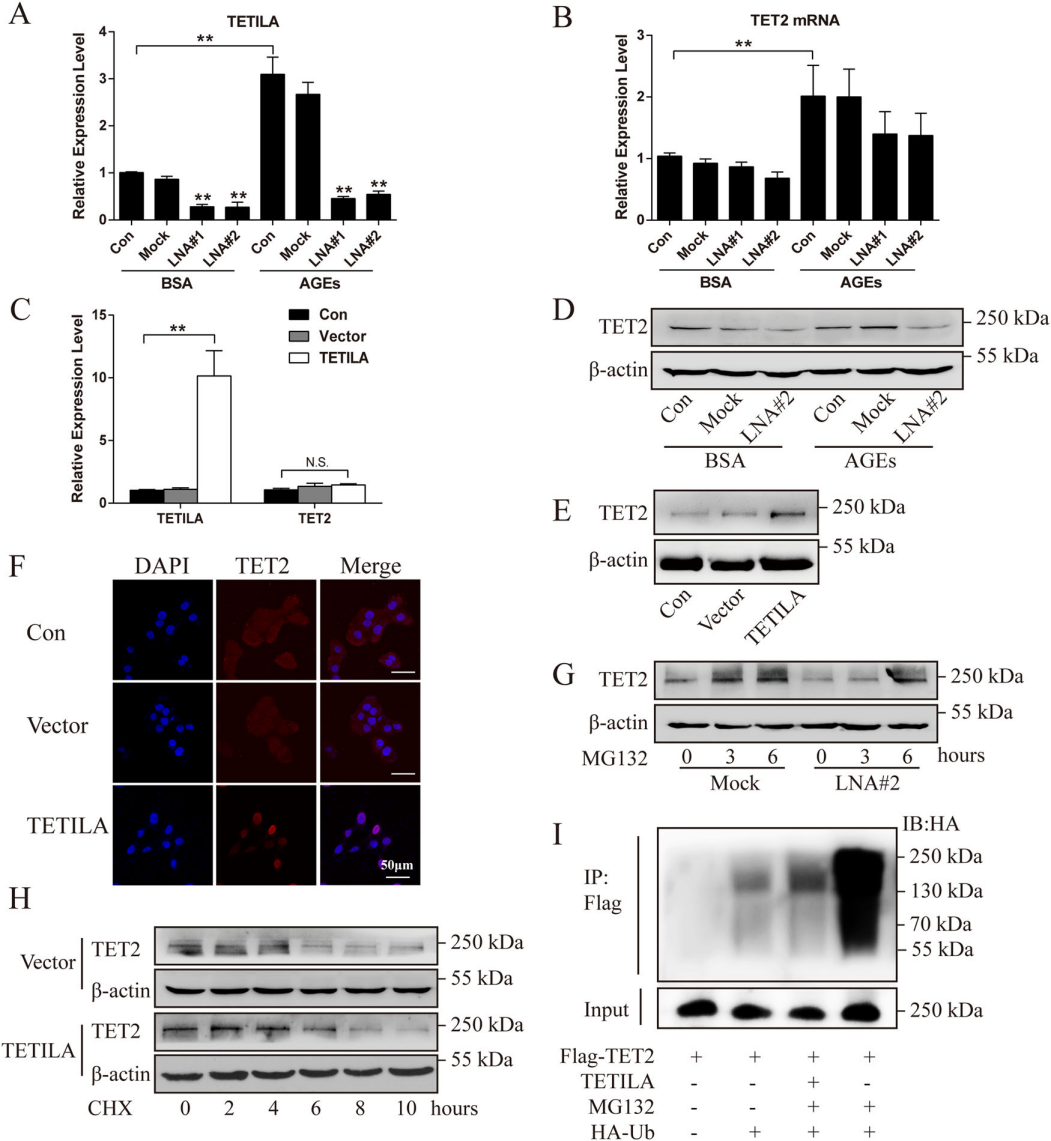
TETILA is associated with TET2 protein expression. **a** TETILA knockdown efficiency measuring using RT-qPCR in HaCaT cells transfected with indicated LNA#1 or LNA#2. **b**, **c** TETILA and TET2 levels were detected by RT-qPCR in HaCaT cells transfected with LNA#1 or LNA#2 followed by stimulation with BSA or AGEs (**b**) or infected with Ad-TETILA or control adenoviruses (Vector) (**c**). **d**, **e** TET2 protein levels detected by western blotting in HaCaT cells transfected with LNA#2 followed by stimulation with BSA or AGEs (**d**) or infected with Ad-TETILA (**e**). **f** Representative confocal images showing the expression and location of TET2 (red) with nuclear staining by DAPI (blue) after infected with Ad-TETILA. **g** TET2 protein levels in HaCaT cells transfected with LNA#2 followed by treatment with MG132 (10 μM) for 0, 3, or 6 h. **h** TET2 protein levels in vector or Ad-TETILA cells treated with CHX (100 μg/ml) for 0, 2, 4, 6, 8 or 10 h. **i** HaCaT cells were transfected with Flag-TET2 and TETILA with or without HA-Ub for 24 h, treated with DMSO or MG132 overnight. Buffers supplemented with or without MG132 (5 μM) were used to lyse cells from each group and to perform IP. Elute were analyzed by IB with HA and Flag antibodies. Data are presented as the mean ± SD of three independent experiments. **P < 0.01 vs. the corresponding control group

We found that neither disrupting TETILA with locked nucleic acid (LNA) (Fig. 2a and S2a) nor an overexpression TETILA-vector (Ad-TETILA) affected TET2 protein expression, but not TET2 mRNA expression (Fig. 2b, c). Knockdown of TETILA with or without AGEs treatment reduced TET2 protein expression (Fig. 2d and S2b), while overexpression of TETILA increased TET2 protein levels (Fig. 2e) and promoted TET2 nuclear translocation (Fig. 2f).

It was previously reported that TET protein stability may be regulated by the ubiquitin-proteasome pathway^22,23^, and many lncRNAs regulate the stability of binding proteins^24–26^. To test whether TETILA affected TET2 protein stability in cytoplasm, we treated HaCaT cells with 10 μM proteasome inhibitor (MG132) after knockdown of TETILA. MG132 treatment for 3 h increased TET2 protein levels, while TET2 protein levels increased only at 6 h after TETILA knockdown (Fig. 2g and S2c). We also treated cells expressing ectopic TETILA with a protein synthase inhibitor (Cycloheximide, CHX) for different incubation times. In the vector group, CHX treatment significantly decreased TET2 protein expression at 6 h. Alternatively, TET2 protein was reduced at 8 h in HaCaT cells overexpressing TETILA (Fig. 2h). Next, we detected TET2 ubiquitination, which is enhanced upon MG132 treatment (Fig. 2i, lanes 4). After TETILA overexpression, while significantly reduces TET2 ubiquitination (Fig. 2i, lanes3).

### TETILA is a positive regulator of TET2 activity

**Fig. 3:**
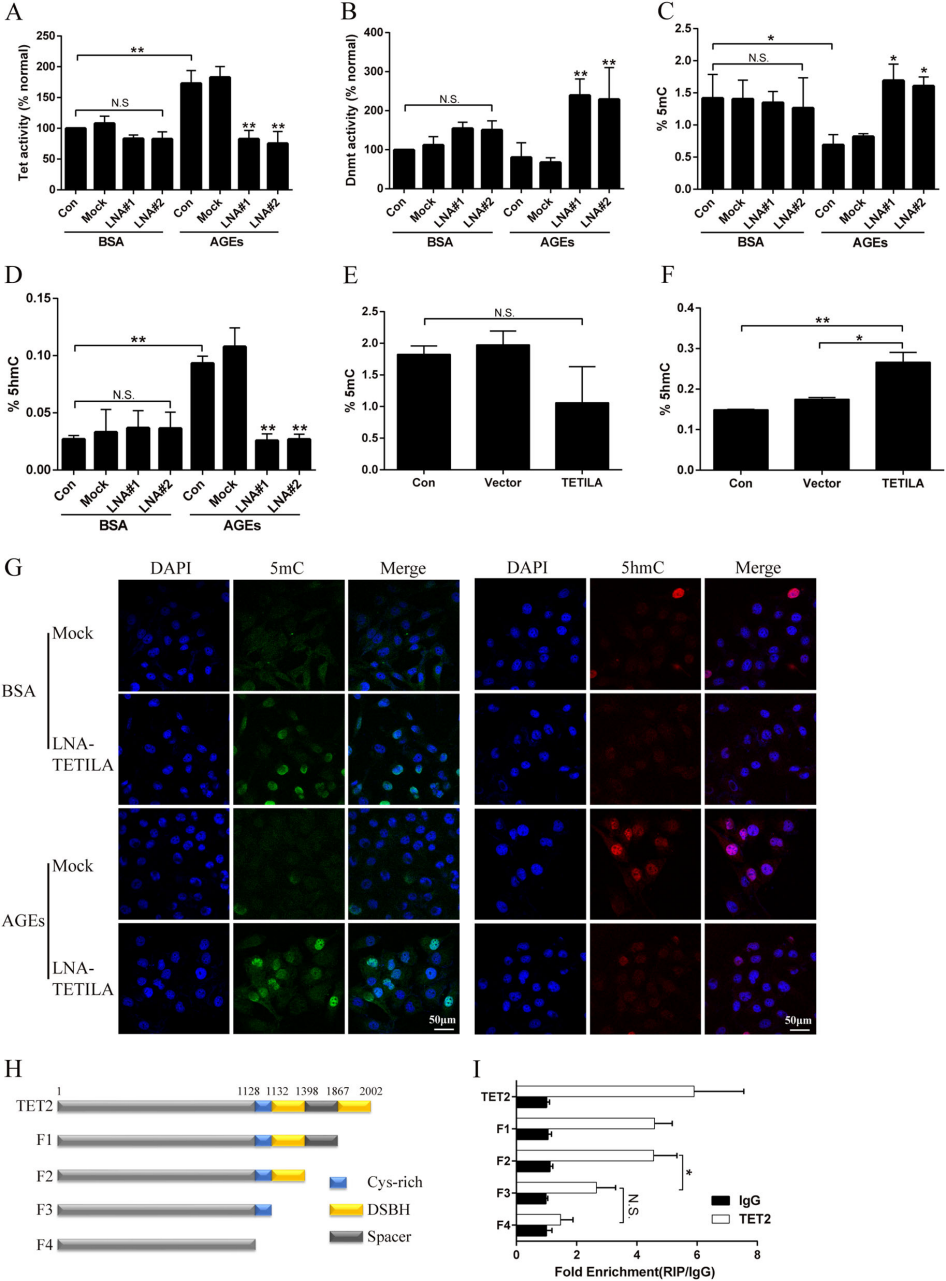
TETILA mediates TET2 protein activity. **a**, **b** Quantification of TET (**a**) or DNMT (**b**) activity assayed by ELISA in HaCaT cells transfected with LNA#1, LNA#2 or Mock and treated with BSA or AGEs. **c**, **d** Quantification of 5mC (**c**) or 5hmC (**d**) activity in HaCaT cells transfected with LNA#1, LNA#2 or Mock followed by treatment with or without AGEs. **e**, **f** Quantification of 5mC (**e**) or 5hmC (**f**) activity in HaCaT cells infected with Ad-TETILA or Vector, examined by ELISA. **g** Confocal images showed the expression of 5mC (green) and 5hmC (red), with nuclear staining by DAPI (blue) after transfection with LNA#2 or Mock. **h** Schematic representation of the different TET2 truncations. **i** HaCaT cells were transfected with various TET2 truncation constructs shown in (**h**) and the cell lysates were analyzed by RIP-qPCR assays (*P* = 0.031). Data are presented as the mean ± SD of three independent experiments. *P *<* 0.05, **P *<* 0.01 vs. the corresponding control group

To further elucidate the role of TETILA in TET activity, we extracted nuclear proteins from cells transfected with scrambled sequence or LNA against TETILA and treated with BSA or AGEs. TET activity in the AGEs-treated group was significantly higher than the BSA group (Fig. 3a), while DNMT activity was not different (Fig. 3b). Silencing TETILA in HaCaT cells significantly decreased TET activity and 5hmc expression in the AGEs-treated group but not the BSA group (Fig. 3a, d, g). Alternatively, silencing TETILA increased DNMT activity and 5mC expression in the AGEs-treated group (Fig. 3b, c and g).

Exogenous overexpression of TETILA increased 5hmC levels (Fig. 3f) but did not change 5mC content (Fig. 3e). Additionally, TETILA had no effect on the 5mC or 5hmC expression in BSA-treated HaCaT cells. We also examined the role of TETILA in TET1 or TET3 expression and found inhibition of TETILA increased TET1 expression but did not affect TET3 expression (Fig. S2d). However, overexpression of TETILA had no effect on TET1 and TET3 protein level (Fig. S2e). It suggests TET1 play an important role in TET activity after knockdown of TETILA in BSA-treatment cells.

To further explore the mechanism of TETILA in the catalytic oxidation of TET2, we truncated different domains of the TET2 protein (Fig. 3h) and overexpressed these different truncation vectors in HaCaT cells. RIP assays revealed that truncating one C-terminal DSBH domain did not affect TET2 binding to TETILA, but truncation of two DSBH domains significantly decreased TET2 enrichment of TETILA fragments. Truncating the domain of Cys-rich and DSBH did not reduce the enrichment of TET2 (Fig. 3i).

### TETILA mediates MMP-9 transcription

**Fig. 4:**
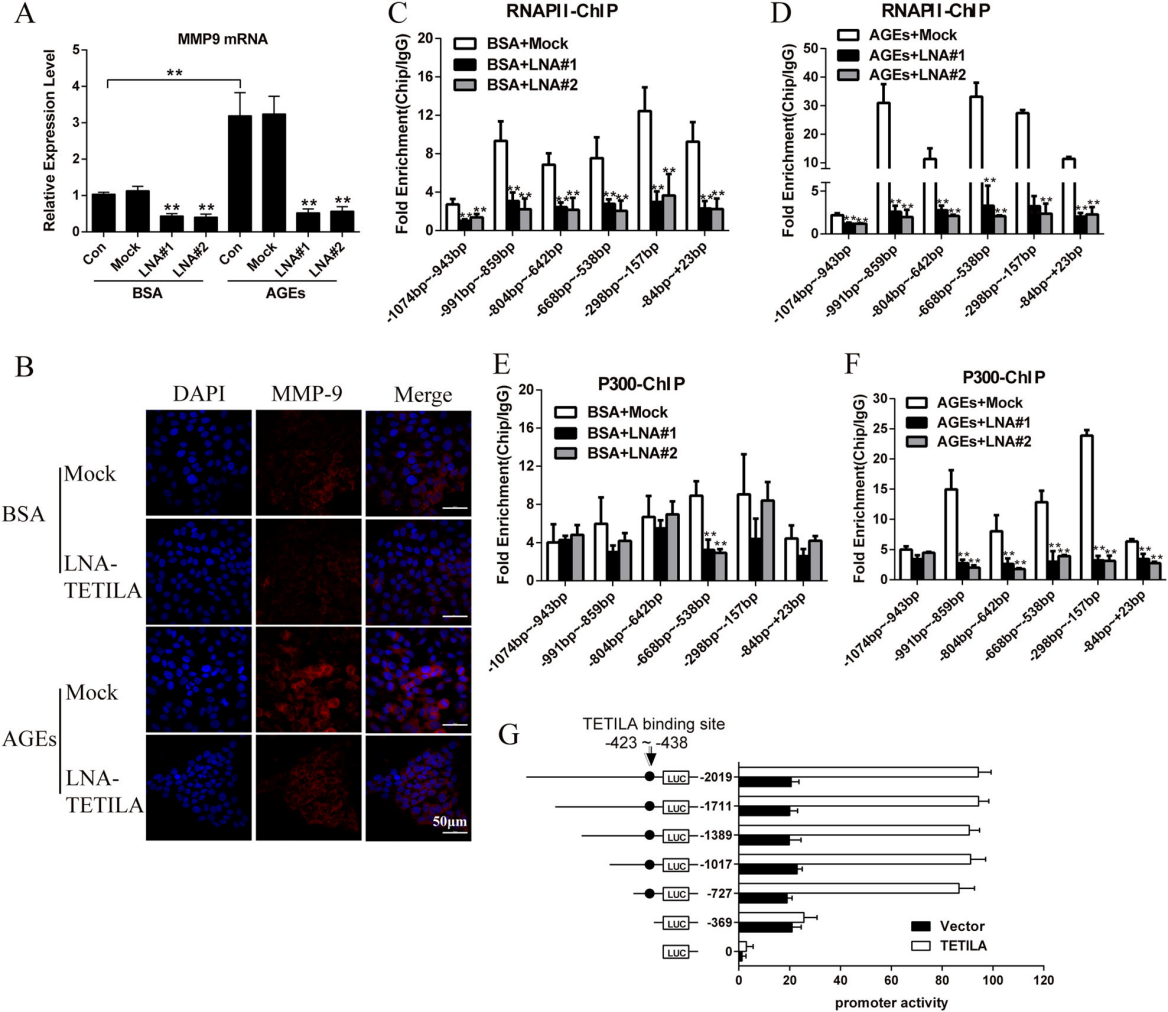
TETILA regulates MMP-9 gene expression. **a** Relative levels of MMP-9 mRNA assayed by RT-PCR in HaCaT cells transfected with LNA#1 or LNA#2 and treated with BSA or AGEs. **b** Representative microscopy images showing the expression of MMP-9 (red) with nuclear staining by DAPI (blue) in HaCaT cells transfected with LNA#2 and treated with BSA or AGEs for 72 h. **c–f** ChIP assay showing RNAP II (**c**, **d**) and p300 **(e**, **f**) occupancy at the MMP-9 locus after knockdown of TETILA in BSA or AGEs-treated HaCaT cells. **g** Luciferase reporter assays for HaCaT cells transfected with reporter plasmids containing truncated MMP-9 promoters and transfected with TETILA or vector for 24 h. Data are presented as the means ± SD of three independent experiments. **P *<* 0.01 vs. the corresponding control group

We found MMP-9 expression was positively correlated with TETILA expression in human skin tissue with diabetes (*n* = 10) and non-diabetes (*n* = 10) (*P* = 0.040, Fig. S3a-c). Knockdown of TETILA decreased MMP-9 expression (Fig. 4a) in both BSA- and AGEs-treated HaCaT cells. This was confirmed by immunofluorescence staining (Fig. 4b). We further investigated the occupancy of RNA polymerase II (RNAP II) and histone acetyltransferase p300 in the MMP-9 promoter using chromatin immunoprecipitation (ChIP) assay. Indeed, knockdown of TETILA in both BSA- or AGEs- treated HaCaT cells decreased the occupancy of both RNAP II (Fig. 4c, d) and histone acetyltransferase p300 (Fig. 4e, f) at the MMP-9 promoter.

To test if TETILA was required for MMP-9 transcription, we next generated a series of PGL4 reporter plasmids containing deleted 5′-flanking regions of the MMP-9 promoter. This revealed that TETILA affected activity of the MMP-9 promoter region from −369 to −727 bp. Therefore, we examined a TETILA binding site located at −423 to −438 bp upstream of the MMP-9 transcription start site (Fig. 4g). As predicted, TETILA critically mediated MMP-9 promoter activity by binding to this region.

### TETILA promotes MMP-9 promoter demethylation

**Fig. 5:**
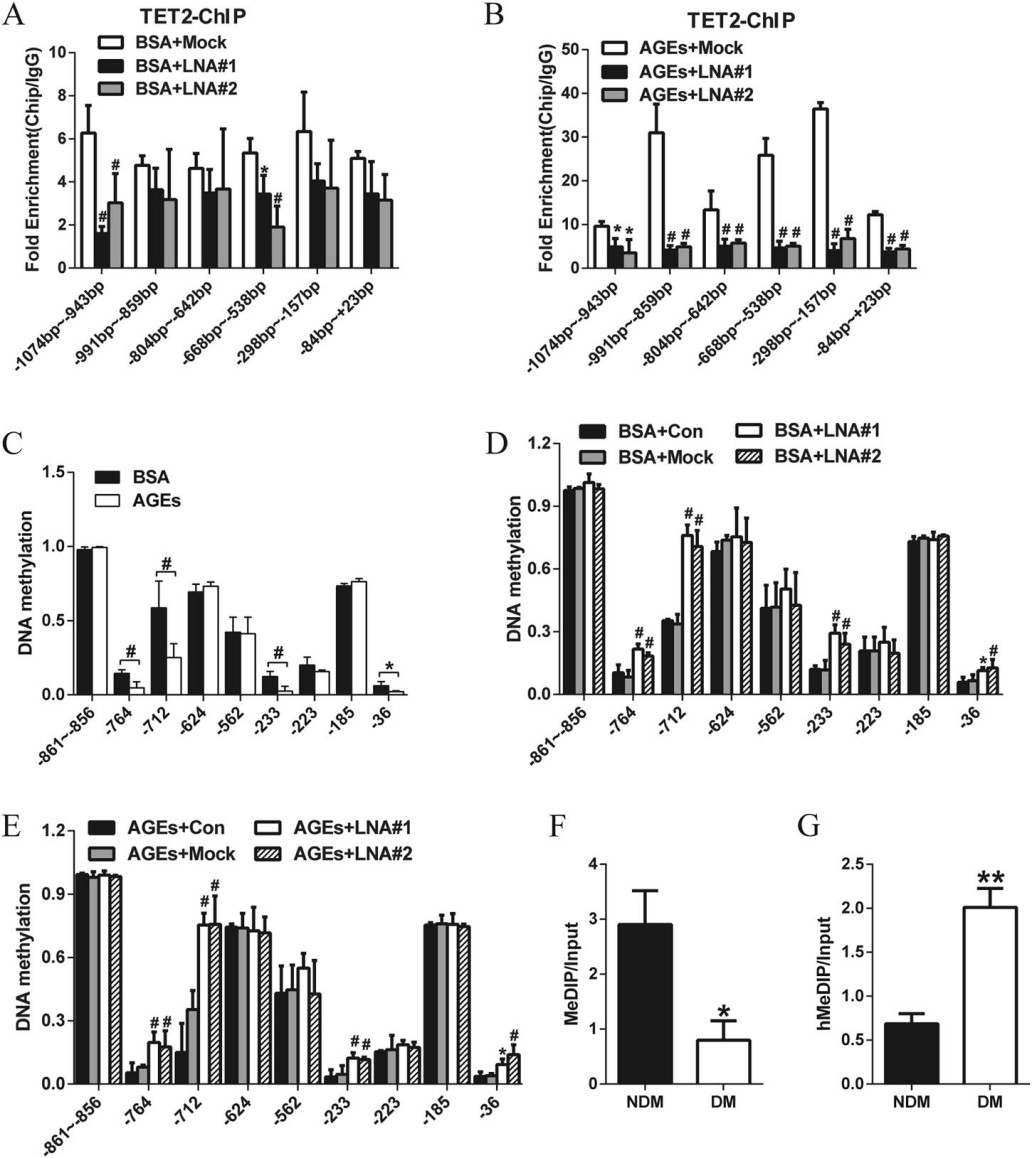
TETILA activates MMP-9 transcription by promoter demethylation. **a**, **b** ChIP assays showed TET2 occupancy at the MMP-9 promoter after knockdown of TETILA in HaCaT cells treated with BSA (**a**) or AGEs (**b**). **c** DNA methylation levels of the MMP-9 promoter assayed by MassARRAY in BSA- or AGEs-treated HaCaT cells. **d**, **e** DNA methylation levels around the TSS of MMP-9-examined by MassARRAY in HaCaT cells transfected with LNA#1, LNA#2 or Mock and treated with BSA (**d**) or AGEs (**e**). **f** MeDIP-qPCR analysis indicated the 5mC enrichment in MMP-9 promoter -668~-538bp region of human diabetic skin tissues (*P* = 0.015). **g** hMeDIP-qPCR analysis for 5hmC enrichment (*P* = 0.004). Data are presented as the mean ± SD of three independent experiments. *P *<* 0.05, ^#^*P* *<* 0.01 vs. the corresponding control group

We knocked down TETILA in HaCaT cells and performed ChIP-PCR to detect the binding of TET2 to MMP-9 promoter region. TETILA knockdown in HaCaT cells abolished the occupancy of TET2 at the MMP-9 promoter following BSA (Fig. 5a) or AGEs (Fig. 5b). To further determine how TETILA affects specific CpG sites, we analyzed 10 CpG sites in the MMP-9 promoter by MassARRAY. AGEs treatment significantly decreased DNA methylation at four sites (−764, −712, −233, and −36 bp, respectively) relative to BSA treatment (Fig. 5c). Intriguingly, TETILA knockdown significantly increased DNA methylation at those four CpG sites in BSA (Fig. 5d) or AGEs-treated cells (Fig. 5e).

To explore the genome regions where TETILA influences MMP-9 expression by modulating 5mC/5hmC levels, we performed methylated DNA Immunoprecipitation (MeDIP) and Hydroxymethylated DNA Immunoprecipitation (hMeDIP) combined with qPCR. We confirmed high TETILA expression could promote 5hmC enrichment in MMP-9 promoter region (Fig. 5f) but inhibit the 5mC enrichment (Fig. 5g).

### TETILA mediates MMP-9 promotor demethylation via the TET-TDG pathway

**Fig. 6:**
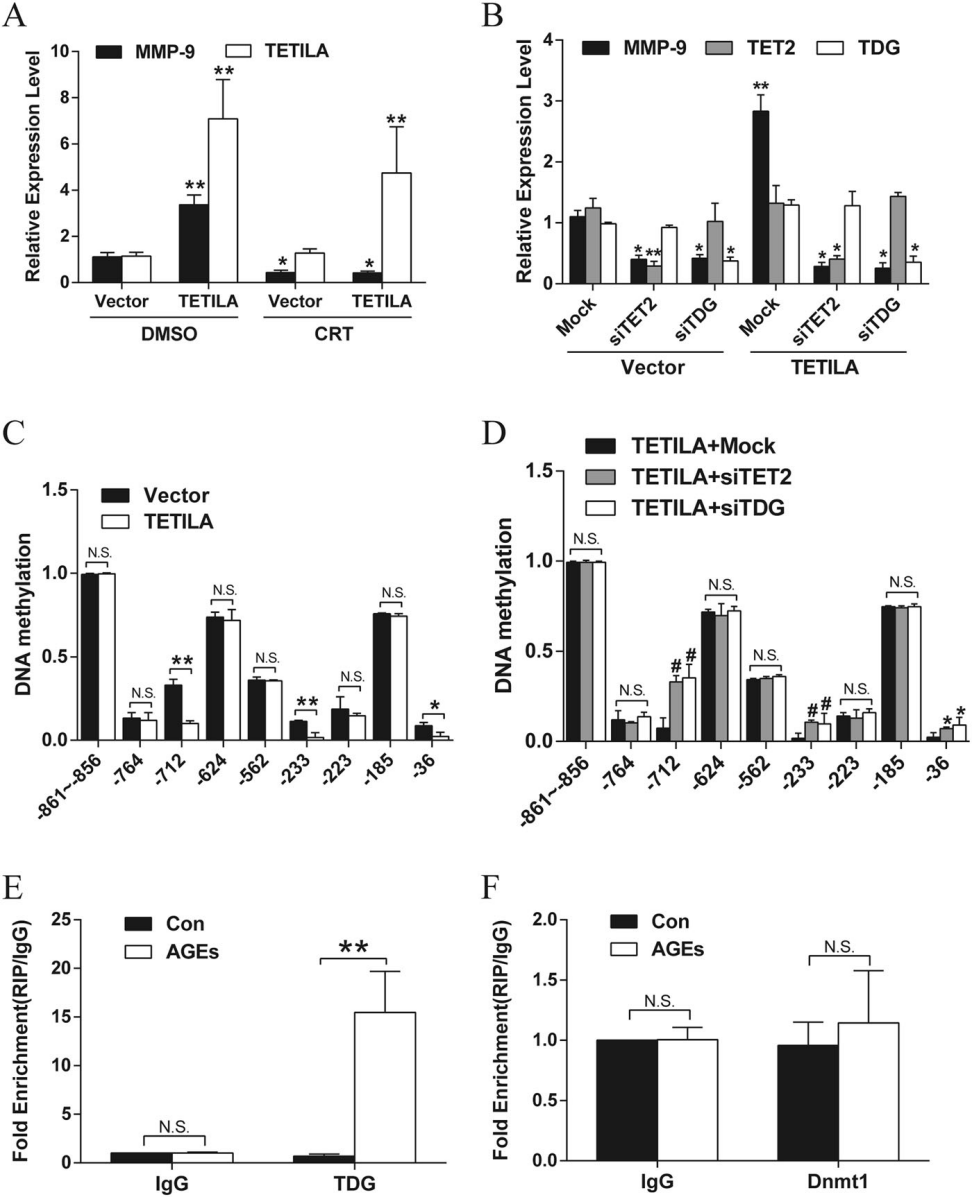
TETILA binds to TET2 and TDG to induce promoter demethylation. **a** Relative levels of MMP-9 in HaCaT cells infected with Ad-TETILA or Vector followed by treatment with the BER inhibitor (CRT) for 48 h. DMSO was used as a solvent control. **b** Relative levels of MMP-9 assayed by RT-qPCR in HaCaT cells infected with Ad-TETILA and co-transfected with siRNA against TDG (siTDG) or TET2 (siTET2). **c**, **d** DNA methylation analysis of the MMP-9 promoter in HaCaT cells after infected with Ad-TETILA (**c**) or TETILA overexpression and transfected with siTDG or siTET2 (**d**). **e**, **f** RIP assays showing interactions between TDG (**e**, *P* = 0.004) or DNMT1 (**f**) and TETILA in HaCaT cells treated with AGEs. Data are presented as the mean ± SD of three independent experiments. *P *<* 0.05, **or ^#^*P* < 0.01 vs. the corresponding control group

Active DNA demethylation refers to an enzymatic process that removes or modifies the methyl group from 5mC. TDG, acting on TET-generated 5fC and 5caC, mediates the first biologically and biochemically validated, complete pathway for active DNA demethylation^27–29^. Our previous study has demonstrated that TDG mediate the demethylation of the MMP-9 promoter. To test the role of TDG in TETILA-induced TET activity, we used RT-qPCR to detect MMP-9 expression after treatment with the BER inhibitor (CRT0044876) and TETILA overexpression. However, treatment with the BER inhibitor significantly reduced MMP-9 levels in both the vector and TETILA overexpression groups (Fig. 6a). Knockdown of TET2 or TDG by siRNA also abrogated the TETILA-induced MMP-9 upregulation in HaCaT cells (Fig. 6b). We then detected the DNA methylation levels of the MMP-9 promoter after overexpression of TETILA in HaCaT cells. Methylation was significantly reduced at three CpG sites in the MMP-9 promoter (−712, −233, and −36 bp, Fig. 6c). However, downregulation of TET2 or TDG inhibited the TETILA-induced demethylation of the MMP-9 promoter (Fig. 6d).

Biochemical and biophysical studies have shown that TET family proteins and TDG physically interact to oxidize and excise 5mC. Specifically, TETs and TDG form stable complexes to induce DNA demethylation^30,31^. DNMT1, a molecule important for maintaining DNA methylation, can interact with TET2 in a “Yin-Yang” complex targeted to chromatin during oxidative stress to prevent DNA methylation^22^. Therefore, we used RIP to explore whether TETILA complexes with TDG or DNMT1. This revealed a combination between TETILA and TDG (Fig. 6e), but not DNMT1 (Fig. 6f). Subsequently, in a coimmunoprecipitation experiment conducted in HaCaT cells, we discovered that ectopic expression of TETILA increased TDG interactions with TET2 (Fig. S3d).

### The role of TETILA in AGEs-treated HaCaT cells

**Fig. 7:**
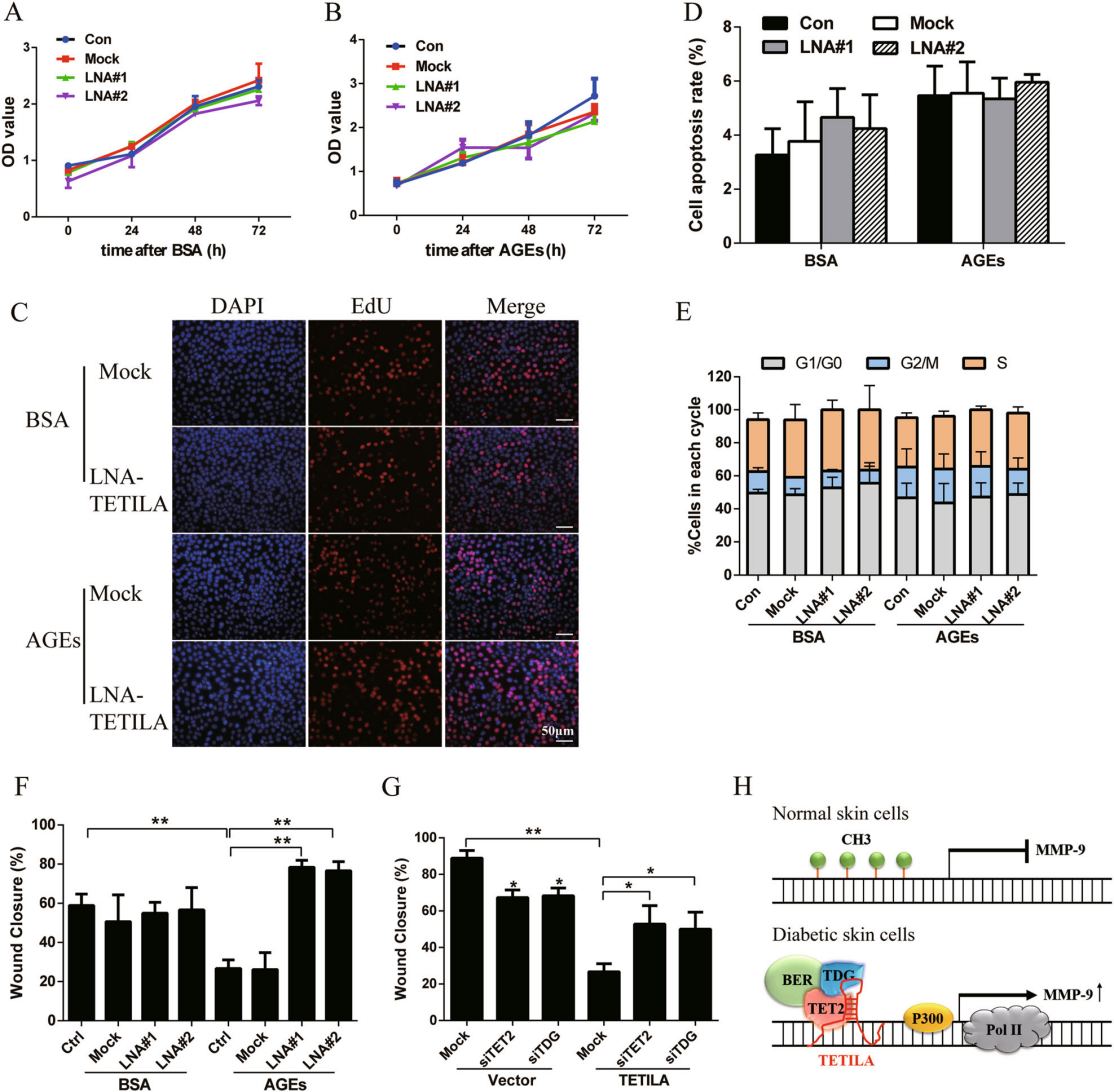
TETILA influences the behavior of AGEs-treated HaCaT cells. **a–c** Proliferation of BSA- or AGEs-treated cells following the downregulation of TETILA (LNA#1 or LNA#2) examined with Cell Counting Kit-8 (**a**, **b**) or EdU assays (**c**). **d** Quantification of the apoptotic rate of the cells. **e** Quantification of the cell -cycle distribution profiles of the cells. **f** Statistical analyses of wound -healing assays in HaCaT cells transfected with LNA#1 or LNA#2. **g** Statistical analyses of wound -healing assays in TETILA overexpressed cells with knockdown of TET or TDG. **h** Model depicting the role of TETILA in the regulation of MMP-9. Data are represented as mean ± SD of three independent experiments. *P < 0.05, **P *<* 0.01 vs. the corresponding control group

To evaluate the biological effects of TETILA, we inhibited TETILA expression in BSA- or AGEs- treated HaCaT cells. Knockdown of TETILA did not affect cell growth or survival, as confirmed by Cell Counting Kit-8 (CCK-8) assays (Fig. 7a, b) and 5-ethynyl-2′-deoxyuridine (EdU) staining (Fig. 7c and S5f). TETILA also did not affect the cell apoptosis (Fig. 7d and S5a) or proportion of cells in various stages of the cell cycle (Fig. 7e and S5b), as determined by flow cytometry. We next investigated the role of TETILA in HaCaT cell migration using in vitro wound healing and transwell assays. Interesting, silencing TETILA promoted cell migration and reversed AGEs-induced wound healing deficits without influencing cell proliferation (Fig. 7f and S5c-e). Conversely, overexpression of TETILA in HaCaT cells apparently reduced cell wound healing in vitro. In addition, inhibition of TET2 or TDG with siRNA can abrogate the role of TETILA in migration (Fig. 7g and S5f), suggesting that TETILA exerts its effect via inducing TET2 and TDG.

## Discussion

This study uncovers a novel finding that a TET2-interacting long noncoding RNA (TETILA) as an important signature in diabetic skin ulcers. We found that TETILA expression was positively correlated with MMP-9 in diabetic skin and inhibiting TETILA downregulated MMP-9 expression, thereby increasing the wound healing ability in vitro. As shown in Fig. 7h, we propose that TETILA promotes TET2 binding to TDG and homes this complex to the MMP-9 promoter. Thus, TETILA could mediate gene-specific demethylation of MMP-9 and leads to refractory diabetic skin ulcers.

Gene demethylation process occurs in a cyclical manner that requires enzymatic activity of multiple proteins^32,33^. Nevertheless, how the protein complex is recruited to specific loci of the MMP-9 promoter remains unclear. One study showed that the lncRNA TARID is a scaffold that is necessary for the assembly of a demethylation protein complex that target specific gene^34^. Our results also indicated that lncRNA TETILA, as a molecular scaffold, interacted with TET2, TDG, and the -423 to -438bp segments of the MMP-9 promoter, ultimately regulating MMP-9 expression. Moreover, inhibiting TETILA did not affect cell proliferation or apoptosis, indicating that TETILA plays a major role in wound healing. Further studies using a large number of clinical samples are necessary to fully illuminate the role of TETILA in diabetic foot ulcers.

A systematic review and meta-analysis shown eight studies in chronic and acute wounds revealed that the most frequently determined matrix metalloproteinases were MMP-2 and MMP-9. In this manuscript, we observed MMP-2 expression levels were increased in AGEs-treated HaCaT cells, but inhibition of TETILA had no effect on MMP-2 expression level (Fig. S3g). Therefore, TETILA maybe mainly regulated the expression of MMP-9 in diabetic wound healing. We also found TETILA levels significantly were increased after 72 h and decreased after 96 h in AGEs-treated group, which was consistent with the MMP-9 demethylation process in our previous study^35^. Thus, TETILA is a key molecule in the demethylation process of MMP-9 promoter demethylation.

MMP-9 is constitutively active, which impairs the balance of ECM synthesis and degradation, and considered to a key factor underlying diabetic wound healing deficits^9,12,36^. Multiple levels regulate MMP-9 expression and activity, such as gene transcription, post-transcriptional processing, and proenzyme activation. MMP9 promoter are rich with transcription factors binding sites, such as FOXO1, SP1, and AP-1, therefore, they involves the process of MMP-9 overexpression^37^. For the first time, we provided the demethylation mechanism showing MMP-9 is activated in the diabetic skin cells. But the function of specific loci in the demethylation of MMP-9 promoter and regulation of transcription factor should be investigated in further studies.

Covalent modification of TET proteins can be regulated by ubiquitylation, which can affect their subcellular localization, chromatin binding, and enzymatic activity^23,38,39^. Our findings showed that TETILA increased TET2 protein stability through the ubiquitin-proteasome pathway. It has been demonstrated that the TET family of enzymes can play overlapping roles in biological process^40^. Our results indicated that knockdown of TETILA increased TET1 expression and potentially involved in TET activity in BSA- treated HaCaT cells. The crystal structure of TET2 protein contains DSBH and Cys-rich domains. The DNA is located above the DSBH core with a methylated cytosine (mC6) flipped out and inserted into the catalytic cavity^41^. We found that TETILA may critically regulate TET2-induced demethylation by binding to the DSBH domain of TET2. Future studies should further investigate the structural mechanism underlying how TETILA modulates TET2.

Transcriptome-wide studies showed that lncRNAs in general exhibit more specific expression profiles than mRNAs; that is, numerous mammalian lncRNAs are expressed in a cell type-, tissue-, developmental stage or disease state- specific manner^42^. We have carried out detailed analyses of human/mouse homology of lncRNA TETILA by analyzing the potential transcripts expressed from the TETILA syntenic region in UCSC genome and could not identify a clear mouse homolog for it. Therefore, we cannot confirm this molecular mechanism in wound healing of diabetic mice in this study, and this is a limitation of our manuscript. Further studies using mouse lncRNA microarray analyze the expression and molecular mechanism of lncRNAs will be necessary to firmly establish the in vitro role for lncRNA in diabetic wound healing.

In conclusion, we demonstrated that the lncRNA TETILA regulates the activity and localization of TET2, establishing a functional link between lncRNA and demethylation proteins through RNA-TET2 interactions. Specifically, TETILA served as a molecular scaffold, providing binding surfaces to assemble the demethylation enzymes TET2 and TDG. This scaffold helped to target this complex to the MMP-9 promoter, promoting MMP-9 demethylation and transcriptional activation. Ultimately, this study demonstrated that TETILA might play a crucial role in diabetic foot ulcer pathogenesis, but TETILA serving as a potential therapeutic target for diabetic skin ulcer needs to be further study. Moreover, DNA demethylation occurs frequently in diabetic tissues and cells, so TETILA-mediated DNA demethylation is relevant to global epigenetic changes during diabetes and its complications.

## Materials and methods

### RNA immunoprecipitation-LncRNA microarray

RNA immunoprecipitation experiments were performed using a Magna RIP RNA-Binding Protein Immunoprecipitation Kit (17–701, Millipore, Billerica, USA) according to the manufacturer’s instructions. Briefly, HaCaT cells were lysed and incubated with protein Sepharose beads, which were conjugated at 4 °C with antibodies against IgG, TET2, TDG, and DNMT1. The immunoprecipitated RNA was purified and detected using lncRNA microarray analysis (Arraystar Human LncRNA Microarray V3; Agilent Technology, Santa Clara, CA), which was performed by Kangcheng Biotechnology Co., Ltd (Shanghai, China).

### Patients specimens

The Institutional Review Board of the Sun Yat-sen Memorial Hospital of Sun Yat-sen University approved the study protocol, which was in accordance with the principles of the Helsinki Declaration II. For the use of these clinical materials for research purposes, samples were collected after receiving written consent and approval from the Institutional Research Ethics Committee. In the present study, subjects with type 2 DM (T2DM) hospitalized for DFU and non-DM were enrolled after obtaining verbal consent. Pregnant women, subjects with diabetes other than type 2, and those with wounds limited to above the ankle joints were excluded. Clinical information for the ten cases of DM skin specimens and ten cases of non-DM skin specimens were presented in Table S3.

### RNA pull-down assay

TETILA was transcribed in vitro using the MEGAscript kit with T7 RNA polymerase (AM1333, Life Technologies) and biotin-labelled with the Biotin RNA Labelling Mix (Pierce, IN, USA). One milligram of whole-cell lysates from HaCaT cells were incubated with 3 µg of purified biotinylated transcripts for 1 h at 25 °C; complexes were isolated with streptavidin agarose beads (Invitrogen). The protein present in the pull-down material was detected by western blot.

### TET or DNMT enzyme activity

HaCaT cells were chilled on ice and processed for extraction of nuclear proteins using the NE-PER Nuclear and Cytoplasmic Extraction Kit (Pierce, Rockforld, IL). The activity of TET or DNMT was assessed in parallel using the Colorimetric TET or DNMT Activity/Inhibition Assay Kit or (P-3086, P-3009, Epigentek, Farmingdale, USA) according to manufacturers’ instructions. Briefly, 10 μg nuclear extracts was added to the sample wells coated with cytosine-rich DNA substrate. Absorbance (optical density) was accessed at 450 nm using a 96-well microplate reader and reported to the negative and positive controls provided by the manufacturer. Fluorescence values were normalized to the amount of protein in each sample.

### Luciferase reporter assays

For the luciferase reporter assays, cells with 70% confluence in 96-well plates were transfected with the indicated luciferase reporters (100 ng) and 1 ng of the pRL-TK Renilla luciferase construct. Forty eight hour after transfection, luciferase activity was measured using the Dual Luciferase Reporter Assay System (Promega) and normalized to Renilla luciferase activity.

### Statistical analysis

All data are presented as mean ± standard deviation (SD) of three independent experiments. The sample size was determined holding the probability of a type-I error at α = 0.05. Statistical analyses were performed using the Statistical Package for the Social Science computer software version 21.0 (IBM SPSS Statistics, Chicago, IL, USA). The Student’s *t*-test was used to compare differences between two groups. Statistical significance was assessed with ANOVA followed by a least significant difference test for multiple comparisons. *P*-value of <0.05 was considered statistically significant.

## Expanded view

Expanded view includes four figures and five tables.

## Supplementary information

Supplemental Materials and Methods

Figure S1

Figure S2

Figure S3

Figure S4

Supplementary Figure Legends

Supplementary Tables

Authors contribution
