# Supplementary material for: TET2-interacting long noncoding RNA promotes active DNA demethylation of the MMP-9 promoter in diabetic wound healing
Source: Cell Death Dis. 2019 Oct 25;10(11):813. doi: 10.1038/s41419-019-2047-6 (PMC6814823; doi:10.1038/s41419-019-2047-6)
Supplement: Supplementary file 1 — Supplemental Materials and Methods [file 41419_2019_2047_MOESM1_ESM.docx]

**Supplemental Materials and Methods**

**Cell Culture.** Primary human keratinocytes were obtained from the hospital during routine infant circumcision[1] and identified as described previously (Supplementary Fig. 1) The immortalized HaCaT cell line was preserved by our laboratory. The cells were cultured in growth medium, containing DMEM (Gibco, Grand Island, NY, USA), supplemented with 10 % fetal bovine serum (FBS, Gibco) and 1 % penicillin/streptomycin (Invitrogen, Garlsbad, CA). Cells became 30~40 % confluence and were treated with 100 μg/ml glycoaldehyde-modified AGE-bovine serum albumin (AGEs, Calbiochem, La Jolla, CA) or bovine serum albumin (BSA, Calbiochem) for 12 to 96 hours.

**Locked Nucleic Acids, siRNAs, Plasmids, Drugs and Antibodies**

Locked nucleic acids (LNAs) against TETILA and scrambled LNA were synthesized by Exiqon and transfected by RNAi Max Transfection Reagents (Invitrogen). siRNAs against TET2, TDG and control siRNA were purchased from Genepharma (Shanghai, China) and transfected with Lipofectamine 3000 reagent (Invitrogen). Primers were synthesized by TianyiBio (Guangzhou, China). Sequences of primers and siRNAs used in this study are listed in Table S4 and Table S5. Plasmids pcDNA-3.1 were purchased from Novagen (Millipore, Billerica), pGL3-basic and pRL-TK were purchased from Promega (Madison, WI).

BER inhibitor CRT 0044876 (CRT) and proteasome inhibitor MG132 were purchased from Sigma-Aldrich (St Louis, MO). Protein synthesis inhibitor Cycloheximide (CHX) was purchased from APExBIO (Houston, Texas). These reagents diluted in dimethyl sulfoxide (DMSO, Sigma, St Louis, MO).

Rabbit polyclonal anti-MMP-9 (ab76003, Abcam, Cambridge, MA), rabbit polyclonal anti-TET1(ABE1034, Millipore, Billerica), rabbit polyclonal anti-TET2 (ab94580, Abcam), rabbit polyclonal anti-TET3 (ab139311, Abcam), anti-5mC (clone 33D3, Active Motif), anti-5hmC (Active Motif), rabbit polyclonal anti-TDG (ab106301, Abcam), mouse monoclonal anti-DNMT1 (ab13537, Abcam), mouse monoclonal anti-RNA polymerase II (RNAP II, ab81, Abcam), rabbit monoclonal anti-KAT3B/p300 (ab106301, Abcam) were purchased. Rabbit monoclonal anti-β-actin (#4970), anti-HA (#3724) and anti-Flag (#14793), Alexa Fluor 488-conjugated anti-mouse secondary antibody (#4408) and Alexa Fluor 555-conjugated anti-rabbit secondary antibody (#4413) were purchased from Cell Signaling Technology (Danvers, MA, USA).

**Plasmids Constructs**

The recombinant adenovirus particles, containing TETILA gene (pHBAD-EF1-MCS-3flag-CMV-mCherry), were constructed by Hanbio Biotechnology (Shanghai, China). A human TET2 cDNA clone encoding transcript variant 1 (2002 aa) was constructed by BersinBio (Guangzhou, China). All TET2 fragments were PCR amplified and cloned into the pcDNA 3.1 vector using BamHI and XhoI (Invitrogen).

**Cell transfection**

Cells were seeded in six-well plates at 40~60 % confluence before transfection. To prepare transfection cocktails for each well, siRNA or LNA (5 μl, with a solution of 20 μM) was mixed with 250 μl Opti-MEM (Invitrogen) by gentle pipetting. In parallel, 5μl of RNAi Max Transfection Reagent or Lipofectamine 3000 was diluted in 250 μl of Opti-MEM by gentle pipetting. After 5 min incubation at room temperature, the two components were combined by gentle pipetting. Following incubation at room temperature for 15 min, the resulting cocktail (500 μl) was directly applied to cells that were equipped with Opti-MEM (1500 μl). After 6 hours incubation in a cell culture incubator, the medium was replaced with fresh medium. RNA and protein were extracted for analysis after 48 hours. HaCaT cells were transfected with TETILA-overexpressing recombinant adenovirus particles (Ad-TETILA) and control adenovirus particles (Ad-vector). Media were replaced with new media 6h after transfection. When the cells were confluent, the cell samples were conventionally dissociated and collected to perform the corresponding experimental detection.

**Fluorescence In Situ Hybridization (FISH)**

For in situ detection of lncRNA-TETILA in HaCaT cells, the probes were designed and produced by Exiqon (TAGTACTCAGCACCTCATTGCA, Vedbaek, Danmark). Cells were briefly rinsed in PBS and fixed in 4 % formaldehyde in PBS for 15min at room temperature. Then the cells were permeabilized in PBS containing 0.5 % Triton X-100 (Sigma) for 15min on ice, washed with PBS 5min three times and rinsed once in 2 × SSC prior to hybridization. Hybridization were performed in hybridization solution (TETILA probe dilution 1:1000, BersinBio, Guangzhou, China) for 16 hours at 37 °C. Cells were washed twice for 5 min in 50 % deionized formamide / 2 × SSC at 53 °C. Then cells were washed in 2 × SSC at 42 °C for 5min and in 0.5 × SSC for 5 min. After washing with PBS, cells were stained with 4,6-diamidino-2-phenylindole (DAPI, Sigma-Aldrich), and imaged using a confocal laser-scanning microscope (Carl Zeiss, Oberkochen,).

**In situ hybridization (ISH) and data analyses**

TETILA expression in paraffin-embedded sections were examined using *in situ* hybridization (ISH). Briefly, after dewaxing and rehydration, the samples were digested with proteinase K, fixed in 4 % paraformaldehyde, hybridized with the 5’digoxin-labeled LNA^TM^-modified TETILA probe (Exiqon) at 55 °C overnight overnight, and subsequently incubated overnight at 4 °C with anti-digoxin monoclonal antibody (Roche, Basel, Switzerland). Then the sections were stained with nitro blue tetrazolium/5-bromo-4-chloro-3-indolylphosphate (NBT/BCIP, Beyotime), mounted and examined.

The staining scores were determined based on both the intensity and proportion of TETILA-positive cells in 10 random fields under a 40× objective. Scoring was conducted according to the immune reactive score (IRS) standard, IRS= proportion of positively stained cells (P)×staining intensity (I). The proportion of positively stained cells: P=0, no positive cells; P = 1, 1~24 % positive cells; P = 2, 25 ~ 49 % positive cells; P = 3, 50 ~ 74 % positive cells; and P = 4, 75 ~ 100 % positive cells. The cells at each staining intensity were recorded as follows: I = 0, no staining; I = 1, light staining intensity; I = 2, moderate staining intensity; and I = 3, dark staining.

**Protein stability assay**

Cells were seeded in a 6-well plate one day before experiments. After 24 hours, fresh medium with 100 μg/ml cycloheximide (CHX, Sigma) was added to cells which were incubated and harvested at indicated time points. Cell pellets were stored at -80 °C until all samples were collected. One well of cells immediately before adding CHX were harvested as control (0 hour). Cell pellets were lysed in RIPA lysis (Beyotime, Shanghai, China) and subjected to standard SDS-PAGE and western blot analysis using anti-TET2 (1:1000 dilution).

**TET or DNMT enzyme activity**

HaCaT cells were cultured for 72 hours under BSA or AGEs with LNA interference TETILA, chilled on ice and processed for extraction of nuclear proteins using the NE-PER Nuclear and Cytoplasmic Extraction Kit (Pierce, Rockforld, IL). The activity of TET or DNMT was assessed in parallel using the Colorimetric TET or DNMT Activity/Inhibition Assay Kit or (P-3086, P-3009, Epigentek, Farmingdale, NY) according to manufacturers’ instructions. Briefly, 10 μg nuclear extracts was added to the sample wells coated with cytosine-rich DNA substrate. Absorbance was accessed at 450 nm using a 96-well microplate reader and reported to the negative and positive controls provided by the manufacturer. Fluorescence values were normalized to the amount of protein in each sample.

**5mC or 5hmC ELISA**

The levels of 5mC and 5hmC were measured according to our previously described protocol[1]. Genomic DNA was diluted to 1ng/ul, added to 96-well microplates (100 ng per well). Unbound DNA was washed off with PBST (0.1% Tween 20), and plates were blocked with 2% BSA in PBST for 2 hours at room temperature (RT), incubated with primary antibody for 30 min at RT, washed, and incubated with secondary antibody for 30min at RT. After wash, TMB substrate was added and absorbance was measured at 450 nm.

**MeDIP/ hMeDIP-qPCR**

DNA methylation levels of diabetic and non-diabetic skin tissues were analyzed by using methylated/hydroxymethylated DNA and Immunoprecipitation kits (MeDIP/hMeDIP kits, Epigentek, Farmingdale, NY). 5mC and 5hmC were captured using a high affinity 5mC antibody and 5hmC monoclonal antibody respectively, and semi-quantitative PCR was performed on DNA eluted from the plate using primers for MMP-9 promoter region (Forward-GCTGCTACTGTCCCCTTTACTGC and Reverse- GCTTCCTCTCCCTGCTTCATCT).

**Cell proliferation assays**

For cell proliferation assays, a total of 3,000 cells were seeded into 96-well plates. After 12 hours of culture, cell proliferation was evaluated using the Cell Counting Kit-8 (CCK8, Dojindo Laboratories, Kumamoto, Japan) according to the manufacturer’s protocol. The cell proliferation curves were plotted using the absorbance at each time point. The 5-Ethynyl-2’-deoxyuridine (EdU) immunofluorescence staining was performed with the EdU Kit (Riobio, Guangzhou, China) according to the manufacturer’s protocol. The results were measured with fluorescence microscope (Nikon, Sendai, Japan) and quantified with Image-J software.

**Immunofluorescence, western blotting, quantitative DNA methylation analysis, chromatin immunoprecipitation assay, immunohistochemistry, cell cycle, apoptosis and wound-healing assay**

For further details, these were measured according to our previously described protocols[2].

**References**

1. Zhang J, et al. AGE-induced keratinocyte MMP-9 expression is linked to TET2-mediated CpG demethylation. Wound Repair Regen, 2016, 24:489-500

2. Zhou L, et al. GADD45a Promotes Active DNA Demethylation of the MMP-9 Promoter via Base Excision Repair Pathway in AGEs-Treated Keratinocytes and in Diabetic Male Rat Skin. Endocrinology, 2018, 159:1172-1186
