# Supplementary figures and images for: TET2-interacting long noncoding RNA promotes active DNA demethylation of the MMP-9 promoter in diabetic wound healing
Source: Cell Death Dis. 2019 Oct 25;10(11):813. doi: 10.1038/s41419-019-2047-6 (PMC6814823; doi:10.1038/s41419-019-2047-6)

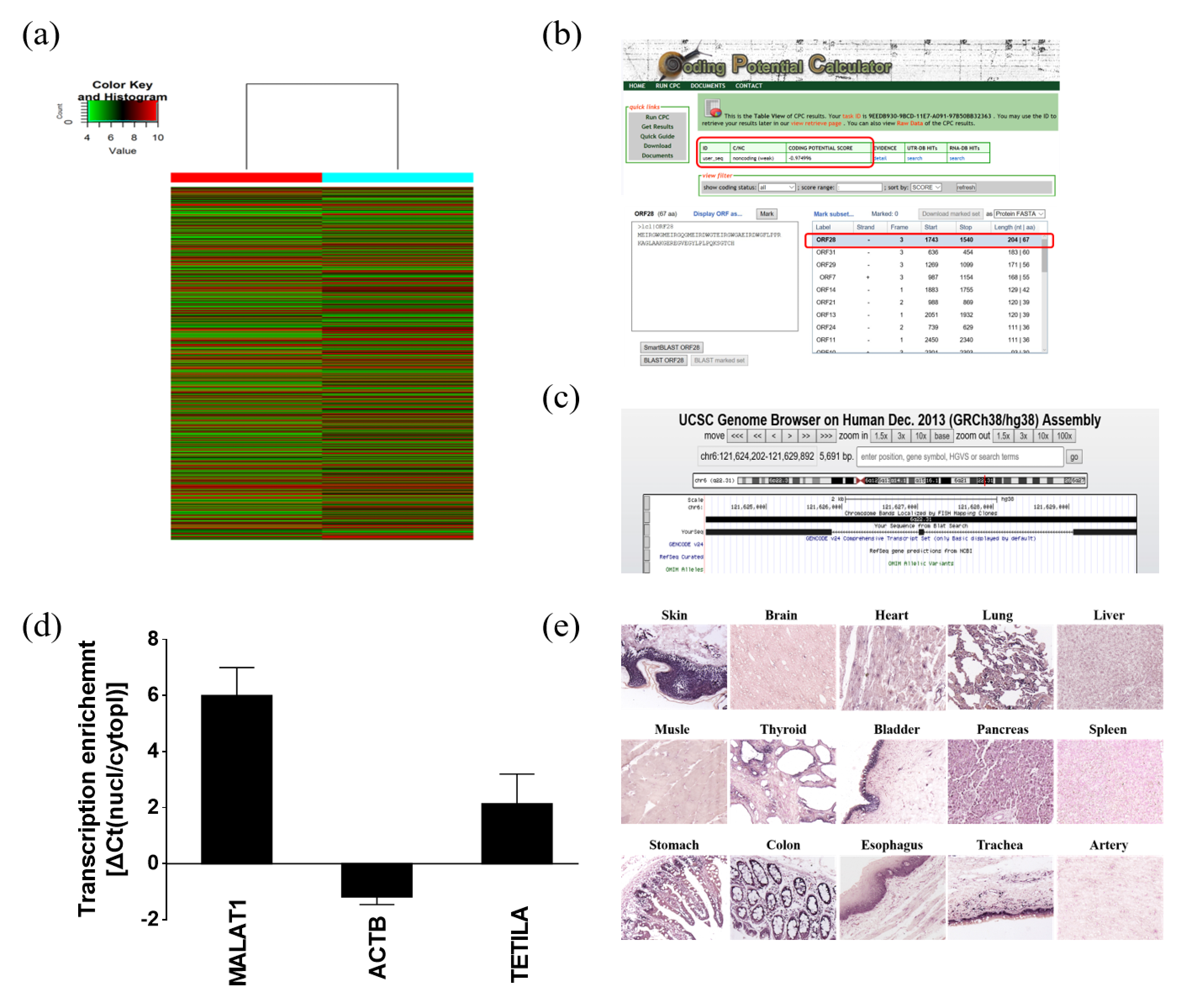

Supplement: Supplementary file 2 — Figure S1 [file 41419_2019_2047_MOESM2_ESM.tif]

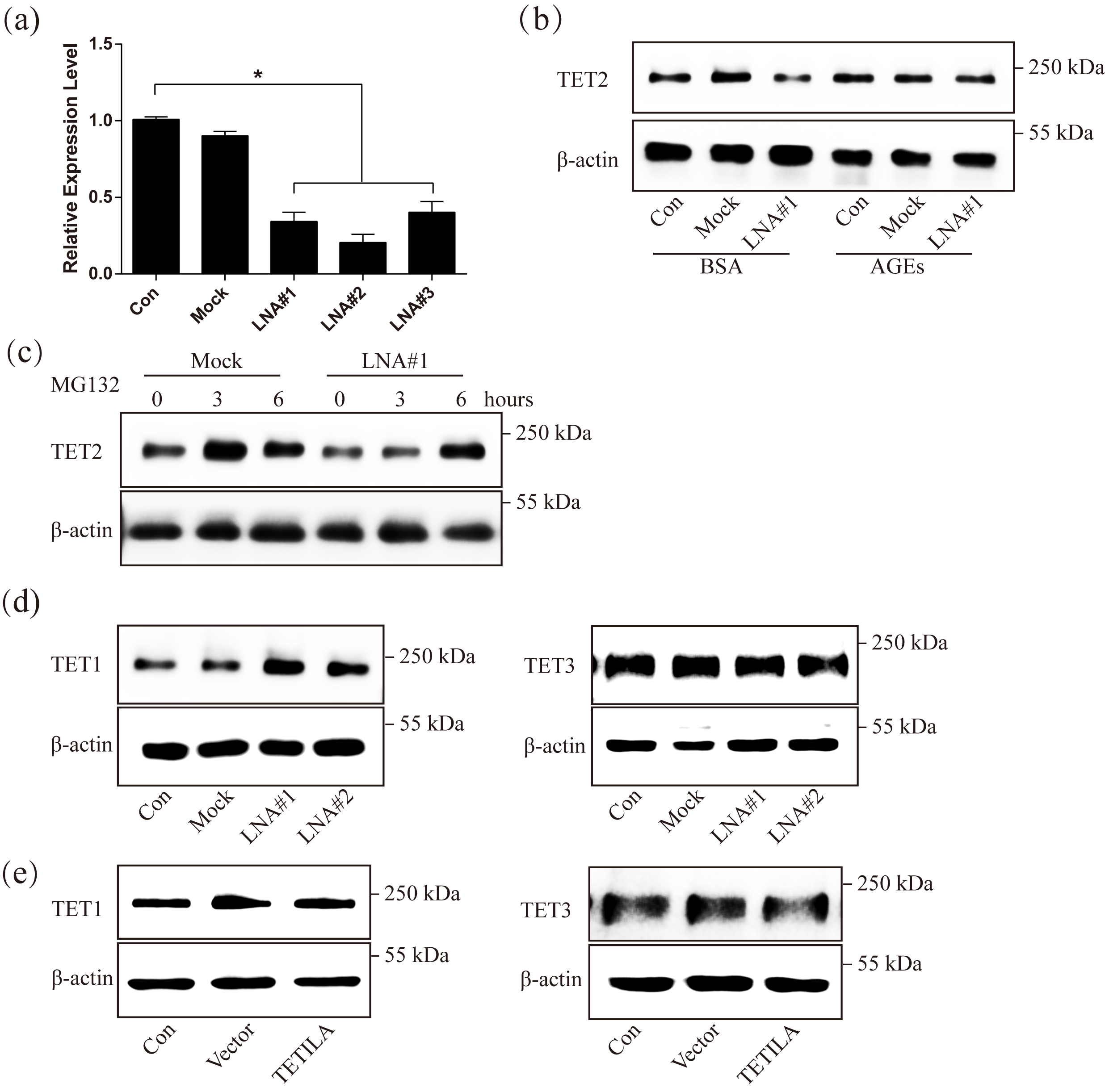

Supplement: Supplementary file 3 — Figure S2 [file 41419_2019_2047_MOESM3_ESM.tif]

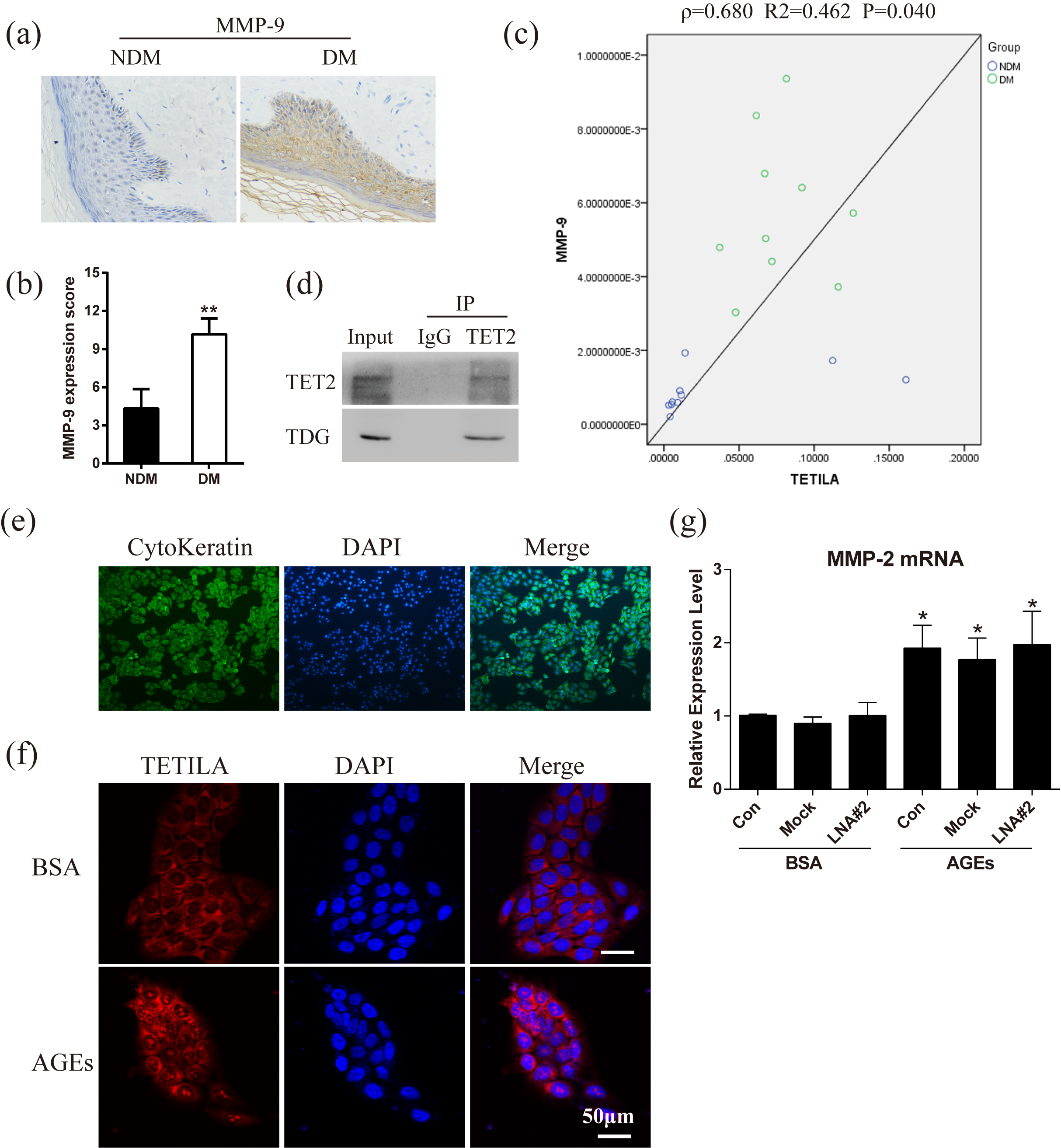

Supplement: Supplementary file 4 — Figure S3 [file 41419_2019_2047_MOESM4_ESM.tif]

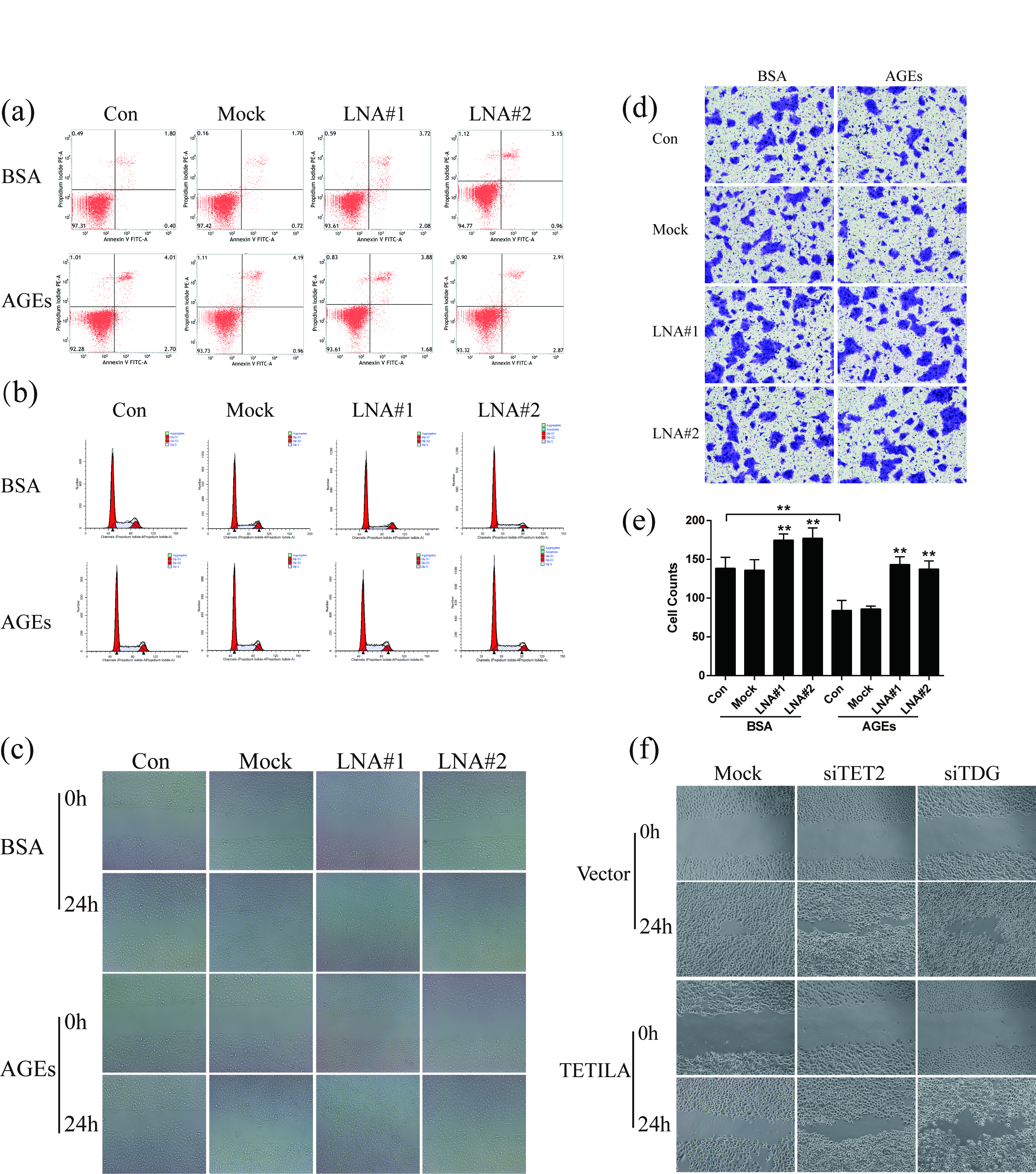

Supplement: Supplementary file 5 — Figure S4 [file 41419_2019_2047_MOESM5_ESM.tif]
