# Supplementary material for: TET2-interacting long noncoding RNA promotes active DNA demethylation of the MMP-9 promoter in diabetic wound healing
Source: Cell Death Dis. 2019 Oct 25;10(11):813. doi: 10.1038/s41419-019-2047-6 (PMC6814823; doi:10.1038/s41419-019-2047-6)
Supplement: Supplementary file 6 — Supplementary Figure Legends [file 41419_2019_2047_MOESM6_ESM.docx]

**Supplementary Figure legends**

**Fig. S1 Differences in lncRNA expression in HaCaT cells.**

**(a)** Heat map representing hierarchical clustering of distinct lncRNA expression profiling in HaCaT cells (fold change >2.0). Red color scale, higher expression; green color scale, lower expression. **(b)** ORF and CPC software predicted the protein-coding potential of TETILA. **(c)** The location of TETILA in genomic. **(d)** The expression of lncRNA, Malat1 and ACTB in nuclear or cytoplasmic fraction in HaCaT cells. **(e)** TETILA levels assessed by ISH in human different tissues.

**Fig. S2 TETILA regulates TET family proteins.**

**(a)** LncRNA TETILA knockdown efficiency in HaCaT cells transfected with indicated LNA (#1-3) by RT-qPCR. **(b)** Western blotting showing TET2 protein levels in HaCaT cells transfected with LNA#1 followed by stimulation with BSA or AGEs. **(c)** Western blotting analysis for TET2 protein levels in HaCaT cells transfected with LNA#1 followed by treatment with MG132 (10μM) for 0, 3, or 6 h. **(d)** Western blotting showing TET1 and TET3 protein levels in HaCaT cells transfected with LNA#1, LNA#2 or Mock followed by stimulation with BSA or AGEs. **(e)** Western blotting showing TET1 and TET3 protein levels in HaCaT cells infected with Ad-TETILA vector. Data represented the mean ± SD for three independent experiments. **P* <0.05 vs the corresponding control group.

**Fig. S3 Correlation between TETILA and MMP-9 expression.**

**(a)** Immunohistochemistry staining of MMP-9 in human skin tissues with diabetes (n=10) or non-diabetes (n=10). **(b)** The histograms indicate the quantitative analysis of MMP-9 expression in patients skin tissues. **(c)** Correlation of TETILA and MMP-9 expression in human diabetic skin tissues (DM, green circles) and normal tissues (Non-DM, blue circles). **(d)** TET2 and TDG were immunoprecipitated from lysates of HaCaT cells transfected with Ad-TETILA. The input sample was 10% of the lysate that was used for IP. **(e)** Immunofluorescence was performed to confirm the identity of isolated skin keratinocytes using antibodies to cytokeratin. **(f)** Confocal FISH images showing nuclear and cytoplasmic localization of TETILA in primary human keratinocytes. **(g)** Relative levels of MMP-2 mRNA assayed by RT-PCR in HaCaT cells transfected with LNA#2 and treated with BSA or AGEs.

**Fig. S4 TETILA affects the biological function of HaCaT cells.**

**(a–b)** Apoptosis **(a)** and cell-cycle **(b)** rate in HaCaT cells with treated with LNA#1 or LNA#2 against TETILA by flow cytometry analysis. **(c)** Representative images of wound-healing assays. HaCaT cells were transiently transfected with LNA#1 or LNA#2 and treated with BSA or AGEs. **(d)** Representative images of transwell assays. **(e)** Statistical analyses of transwell assays. **(f)** Representative images of wound-healing assays in TETILA overexpressed cells with knockdown of TET or TDG. Data are presented as the means±SD of three independent experiments. **P* <0.05 and ***P*<0.01 vs the corresponding control group.
