# Supplementary material for: TET2-interacting long noncoding RNA promotes active DNA demethylation of the MMP-9 promoter in diabetic wound healing
Source: Cell Death Dis. 2019 Oct 25;10(11):813. doi: 10.1038/s41419-019-2047-6 (PMC6814823; doi:10.1038/s41419-019-2047-6)
Supplement: Supplementary file 7 — Supplementary Tables [file 41419_2019_2047_MOESM7_ESM.docx]

**Supplementary Tables**

**Supplementary Table 1. LncRNA expression profile of interacting with TET2 in HaCaT cells by lncRNA microarray.**

**Supplementary Table 2. Sequence of lncRNA TETILA trascript**

TTTAAACCCTAACTTACAGAAGTATAACAAATATTTGTAGGGAAGAAATACTTTGACAGT

GTGTAAAGCAGTAAGACGAGTTTCTTTTCTGTCACATAAATGCCAAAAGTCTTGCAAAAG

TCTTCTGATGATGAGAATCACAGTTTAAAGCTGGGTGGAAACCAAGGCTATATCCTACCC

TGGGAATAGTAGACTTTATCTTCTGATATCTCTCCTGTCCCTGACTGTTGCAATGAGGTG

CTGAGTACTAGCTAGCTATGTAGAAGAGGTTAGGAGATTGGGGTGAAGAATTTATTTAAA

AAGATGCTCTATTGAGAATCTTGGGTCATTACCCCAACAGGTAAAAAATGCATTAAAATC

ATCTATGCTGACATCAATATAGTTTGAAAAAATGTAGATTACCAGGCTGATTTGGATCAC

TCAAAAGCATTGGGTAAACATCTGTGTGTGGCACTACACAAAGGAAACTAAACAGAAGTG

TCCTGCTTCAAGGGCACAATTATAGGCGATTTTTATAAAGGCATTGATACATCCAGGAAG

TCTACGAAAGCCTGCTGGTTCCAGTTTGCCTAAAGTATGAGGGGATTCAGGAAATAAGTT

ACTATACCCTATAAAGTTAGAAGGACCCTTAGGCATCCAGTGAGACTGCCAGTTGAGGAC

TCTGGTTCAGATCAAAAGACATCTGGCAGAAACTGACTGCTAGAAAAGGGGCTGTTGAGG

CTCTTTTTCTAATCCACATTTGAGAGGGCAAATGACAAGCATCCCCTTTGGCTCAAAGGG

GAGAGGCAGAGTCCAGGATGTTTAACATTCATTAGGCGCTAGATACTGAACAGATCAGCT

CATGGATACTCTTCATGATCTGAAGGAACTATTGAACCTCTTCCCAGAGAAAGGCACTCA

CAGACGTGCATGAAATTTGGCGCCGTGACTCGGATTGGGGGAACTCCCCTGGGAGATCAA

TCCCCTGTCCTCTTGTTCTTTGCTCCATGAGAAAGATCCACCTACGACCTCAGGTCCTCA

GACTGACCAGCCCAAGGAACATCTCACCAATTTTAAATCAGGTAGGTGGCCTCTTCTTAC

TCTCTTCTCCAACCTCTCTCACTGTCCCTCAACCACTTTCTTCTTTCCACTCTTCAATCT

CTCCCTTCTCTTAATTTCAATTCCTTTCATTTTCTGGGAGAGACAAAGGAGACACGTTTT

ATCTGTGGACCCAAAACTCCGGCGCCAGTCACAGACTGGGAAGGCAGGCTTCCCTTGGTG

TTTAATCATTGCAGGGATGCCTCTCTGATTATTCACCCACGTTTCAAAGGTGTCAGACCA

CGCAGGGACGCCTGCCTTGGTCCTTCACGCTTAGCAGCAAGTCCCGCTTTTCTGGGGAAG

AGGCAAGTACCCCAACCCCTTCTCTCCTTGTCTCTACCCCTTCTCTCCTTTCCTGGGGCA

GGGGCAAGTACCCCTCAACCCCTTCTCCTTCACCCTTAGTGGCAAGTCCTGCTTTTCTGG

GTGAGGGGCAAGTACCCCTCAACCCCTTCTCCTTCACCCTTAGTGGCAAGTCCCGCTTTT

CTGGGGGAGGGGCAAGTACCCCTCAACCCCTTCTCTTTCACCCTTAGCGGCAAGTCCCGC

TTTTCTAGGGGGCAAGAACCCCCAATCCCTTATTTCCGCACCCCAACCTCGTATCTCTGT

GCCCCAATCCCTTATTTCCATGCCCTGACCCCTTATTTCCATGCCCCAACCCCTTATTTC

CATGCCCTGACCTCTTATCTCTGTGCCCTAACCCCTTTTCCCACTTTTCTGGAAGGGAAG

AACCCCCAAACCCCTTCCCTCCATTTCTCTACTCTCTTCTCTAGGCTTGCTTCCTTCACT

ATAGGCAACCTTCCACCCTCCATTCCTCCTTCTACTCCCTTGGCCTGTGTTCTCAAAAAC

TTAAAATCTCTTCAACTCACAACTGACCTAAAACCTAAATGCCTTATTTTCTTCTGCAAT

GCCACTTGACCCCAATACAAACTCGACTGTAGTTCCAAATAGCCAGAAAATGGCACTTTG

AATTTTTCCATCCTGCAAGATCTAAATAATTCTTGTCCTAAAATAGGCAAACAGTCTGAG

GTGCCTGACGTCCAGGCATTCTTTTACACATCAGTCCCTTCCTTGTCTCTGTGCCCAGTG

CAAATCATCCCAAATCTTCCTTCTTTCCCTCCCGCCTGTCCCCTAAGTCCCAAACCCAAG

CATCGATGAGTCTTTCTAATCTTCCTTTTCTACAGACCCATCTGACCTCTCCCCTCCTTG

CCAGGCCTAGCTAGGTCCCAATGCTTCCTCAGCCTCCGCTCCTCCACCCTATAATCTTTT

TATCGCCTCCCCTCCTCACACCTGGTCTGGCTTACAGTTTCGTTCTTGACTAGCCCTCCC

CCACCTGCCCAGCAATTTACTCTTAAAAAGGTGGCTGGAGCCAAAGGCATAGTCAAGGTT

AATGCTCCTTTTTCTTTATCCCAAATCGGATAGCGTTTAGGCTCTTTTTCATCAAATATA

AAAATCCAGCCCAGTTCATGACTTGTTTGGCAGCAACCCTGAGA

**Supplementary Table 3. Clinical characteristic of participants (mean ± sd).**

| **Participants** | | **DM patients**  **N=10** | **non-DM patients**  **N=10** |
| --- | --- | --- | --- |
| Age (years) |  | 59.5±14.9 | 60.2±9.5 |
| Diabetes duration (years) |  | 9.4±3.1 | - |
| BMI (Kg/m^2^) |  | 24.9±2.9 | 22.4±2.5 |
| FBG (mmol/L) |  | 9.7±2.6 | 5.4±0.7 |
| HbA_1c_ (%)***** |  | 9.4±3.0 | 5.7±0.6 |
| SAP (mmHg) |  | 128.2±19 | 137.0±23.9 |
| DAP (mmHg) |  | 75.2±5.9 | 80.4±10.4 |
| Total cholesterol (mmol/L) |  | 3.1±0.9 | 4.0±0.4 |
| Triglyceride (mmol/L) |  | 1.8±0.7 | 2.0±0.41 |
| HDL-cholesterol (mmol/L) |  | 0.8±0.3 | 1.0±0.3 |
| LDL-cholesterol (mmol/L) |  | 3.2±2.8 | 3.5±1.7 |

DM: Diabetes mellitus; BMI: Body mass index; SAP: Systolic artery pressure; DAP: Diastolic artery pressure; HDL-cholesterol: High-density lipoprotein-cholesterol; LDL-cholesterol: Low-density lipoprotein-cholesterol. **P*=0.008

**Supplementary Table 4. List of primers used for RT-qPCR.**

| Gene | Primer sequence | Length(bp) |
| --- | --- | --- |
| TETILA |  | 520 |
| Forward | CTTTTCTGTCACATAAATGCCAA |  |
| Reverse | AGTAACTTATTTCCTGAATCCCCT |  |
| TET2 |  | 195 |
| Forward | CCCGCTGAGTGATGAGAACA |  |
| Reverse | TGTGCTGCTGAATGTTTGCC |  |
| MMP9 |  | 216 |
| Forward | TCGAACTTTGACAGCGACAAGA |  |
| Reverse | TTCAGGGCGAGGACCATAGA |  |
| TDG |  | 109 |
| Forward | TGACCAAGACTCTCCCCGATA |  |
| Reverse | CCAGGTCCAGGGTAATGATGC |  |
| MMP2  Forward  Reverse  ACTB | CCCACTGCGGTTTTCTCGAAT  CAAAGGGGTATCCATCGCCAT | 89 |
| Forward | GTGGCCGAGGACTTTGATTG | 73 |
| Reverse | CCTGTAACAACGCATCTCATATT |  |

**Supplementary Table 5. Interference sequence of the target genes**

| Gene | Sense | Antisense |
| --- | --- | --- |
| TET2-homo-1404 | CUAGCUGCAAUGCUAAAUATT | UAUUUAGCAUUGCAGCUAGTT |
| TET2-homo-3775 | CUGCUUCUGUUCUCAAUAATT | UUAUUGAGAACAGAAGCAGTT |
| TDG-homo-758 | CACUCUACCAGGGAAGUAUTT | AUACUUCCCUGGUAGAGUGTT |
| TDG-homo-830 | CUCCAGUAAAGAAUUUCGUTT | ACGAAAUUCUUUACUGGAGTT |
| LNA#1 TETILA-2160 | 5’-ATTTGGGATGATTTGC-3’ | |
| LNA#2 TETILA-2375 | 5’-GTCAAGAACGAAACTG-3’ | |
| LNA#3 TETILA-531 | 5’-CGTAGACTTCCTGGAT-3’ | |
