# Supplementary material for: TET2-interacting long noncoding RNA promotes active DNA demethylation of the MMP-9 promoter in diabetic wound healing
Source: Cell Death Dis. 2019 Oct 25;10(11):813. doi: 10.1038/s41419-019-2047-6 (PMC6814823; doi:10.1038/s41419-019-2047-6)
Supplement: Supplementary file 8 — Authors contribution [file 41419_2019_2047_MOESM8_ESM.pdf]

DECLARATION OF CONTRIBUTIONS TO ARTICLE

ADMC

Manuscript Number: CDDIS-19-2263R

Journal Name: *Cell Death & Differentiation* (the 'Journal')

Proposed Title of the Contribution: (the 'Contribution)

Author(s): (the 'Authors')

Liyan Zhou, Meng Ren, Tingting Zeng, Wei Wang, Xiaoyi Wang, Mengdie Hu, Shicheng Su, Kan Sun, Chuan Wang, Jing Liu, Chuan Yang, Li Yan

For all *CDD* articles, each person named as an author in the published version must be able to show he or she has contributed substantially to the article.

Authorship credit should be based on 1) substantial contributions to conception and design, acquisition of data, or analysis and interpretation of data; 2) drafting the article or revising it critically for important intellectual content; and 3) final approval of the version to be published. Authors should meet conditions 1, 2 and 3.

Any person who cannot be shown to have made a substantial contribution to the article cannot be listed as an author in the final version. The name of any person who is deemed to have made a minor contribution can, however, appear in the Acknowledgments section of the article.

Please complete the table below to indicate the contributions of all named authors to the manuscript.

Author Full Name:	Specification of Contribution to the Manuscript:
Liyan Zhou	Contribution to the conception and design of the study; acquisition, analysis, and interpretation of the data; and drafting and revising of the article.
Meng Ren	Contribution to the conception and design of the study; acquisition, analysis, and interpretation of the data; and drafting and revising of the article.
Tingting Zeng	Collection of clinical data and the measurement of TETILA expression in diabetic skin; and drafting the article.
Wei Wang	Collection of clinical data and the measurement of TETILA expression in diabetic skin; and drafting the article.
Xiaoyi Wang	Collection of associated data and performed the molecular biological experiments; and drafting the article..
Mengdie Hu	Collection of associated data and performed the molecular biological experiments; and drafting the article..
Shicheng Su	Acquisition of data and critical revision of the manuscript, and revising the article.
Kan Sun	Collection of associated data and performed the molecular biological experiments; and drafting the article..
Chuan Wang	Collection of associated data and performed the molecular biological experiments; and drafting the article..
Jing Liu	Collection of clinical data and the measurement of TETILA expression in diabetic skin; and drafting the article.
Chuan Yang	Guarantors of this work and had full access to all of the data in the study; they take responsibility for the integrity of the data and the accuracy of the data analysis.
Li Yan	Guarantors of this work and had full access to all of the data in the study; they take responsibility for the integrity of the data and the accuracy of the data analysis.

Please complete the table below to indicate the contributions of all named authors to the figures.

Figure 1:

Liyan Zhou, Meng Ren, Tingting Zeng, Jing Liu and Wei Wang worked on the production of this figure. Liyan Zhou and Jing Liu provided data; Meng Ren and Wei Wang made statistical analysis of data; Tingting Zeng made the subset of this figure; Liyan zhou organized the whole figure.

Figure 2:

Liyan Zhou, Meng Ren, Xiaoyi Wang and Mengdie Hu worked on the production of this figure.
Xiaoyi Wang and Mengdie Hu performed the collection of experimental data; Meng Ren made statistical analysis of data; Liyan Zhou made the figure.

Figure 3:

Liyan Zhou, Chuan wang, Kan Sun and Meng Ren worked on the production of this figure. Liyan Zhou provided experimental data and pictures; Chuan wang and Kan Sun made statistical analysis of data; Liyan zhou and Meng Ren organized the whole figure.

Figure 4:

Liyan Zhou, Meng Ren, Wei Wang and Shicheng Su worked on the production of this figure. Liyan Zhou performed the collection of experimental data; Wei Wang and Shicheng Su makes statistical analysis of data and check the figure; Liyan Zhou made the figure.

Figure 5:

Liyan Zhou, Meng Ren, Tingting Zeng, Wei Wang worked on the production of this figure. Liyan Zhou and Tingting Zeng performed the collection of experimental data; Wei Wang and Meng Ren made statistical analysis of data and check the figure; Liyan Zhou and Meng Ren made the figure.

Figure 6:

Liyan Zhou, Meng Ren and Tingting Zeng worked on the production of this figure. Liyan Zhou performed the collection of experimental data; Meng Ren and Tingting Zeng made statistical analysis of data and check the figure; Liyan Zhou made the figure.

Signed for and on behalf of the Author(s):	Print Name:	Date:
Liyan Zhou	Liyan Zhou	3rd October, 2019
